# Glioma Specific Extracellular Missense Mutations in the First Cysteine Rich Region of Epidermal Growth Factor Receptor (EGFR) Initiate Ligand Independent Activation

**DOI:** 10.3390/cancers3022032

**Published:** 2011-04-18

**Authors:** Susie I. Ymer, Sameer A. Greenall, Anna Cvrljevic, Diana X. Cao, Jacqui F. Donoghue, V. Chandana Epa, Andrew M. Scott, Timothy E. Adams, Terrance G. Johns

**Affiliations:** 1 Oncogenic Signaling Laboratory, Monash Institute of Medical Research, Monash University, Clayton, VIC 3168, Australia; E-Mails: ymersi@gmail.com (S.I.Y.), sameer.greenall@csiro.au (S.A.G.), acurljev@btk.fi (A.G.); 2 Tumor Targeting Laboratory, Ludwig Institute for Cancer Research, Heidelberg, VIC 3084, Australia; E-Mails: diana.cao@ludwig.edu.au (D.X.C.), andrew.scott@ludwig.edu.au (A.M.S.); 3 CSIRO Division of Materials Science and Engineering, Parkville, VIC 3052, Australia; E-Mails: vidana.epa@csiro.au (V.C.E.); tim.adams@csiro.au (T.E.A.)

**Keywords:** EGFR, de2-7EGFR, extracellular domain mutation, autoactivation, dimerization, disulfide bond, free cysteine

## Abstract

The epidermal growth factor receptor (EGFR) is overexpressed or mutated in glioma. Recently, a series of missense mutations in the extracellular domain (ECD) of EGFR were reported in glioma patients. Some of these mutations clustered within a cysteine-rich region of the EGFR targeted by the therapeutic antibody mAb806. This region is only exposed when EGFR activates and appears to locally misfold during activation. We expressed two of these mutations (R324L and E330K) in NR6 mouse fibroblasts, as they do not express any EGFR-related receptors. Both mutants were autophosphorylated in the absence of ligand and enhanced cell survival and anchorage-independent and xenograft growth. The ECD truncation that produces the de2-7EGFR (or EGFRvIII), the most common EGFR mutation in glioma, generates a free cysteine in this same region. Using a technique optimized for detecting disulfide-bonded dimers, we definitively demonstrated that the de2-7EGFR is robustly dimerized and that ablation of the free cysteine prevents dimerization and activation. Modeling of the R324L mutation suggests it may cause transient breaking of disulfide bonds, leading to similar disulfide-bonded dimers as seen for the de2-7EGFR. These ECD mutations confirm that the cysteine-rich region of EGFR around the mAb806 epitope has a significant role in receptor activation.

## 1. Introduction

The epidermal growth factor receptor (EGFR) belongs to the ErbB family of receptor tyrosine kinases (RTKs) that also include ErbB2, ErbB3 and ErbB4 [1]. EGFR can bind at least seven ligands, of which the most widely studied is epidermal growth factor (EGF) [1]. Regulated activation of the EGFR requires high affinity ligand binding and involves homo- and/or hetero-dimerization with other ErbB family members [2]. Dimerization is essential for activation of its intrinsic kinase activity and subsequent autophosphorylation [3-6]. These events activate a number of different downstream signaling pathways that regulate multiple cellular processes such as proliferation, differentiation and development. The EGFR is commonly overexpressed or mutated in many cancer types and its presence promotes tumor progression and survival.

In glioblastoma multiforme (glioma), a highly malignant and lethal neoplasm of the brain, amplification of the EGFR gene leads to overexpression of the receptor and is associated with a number of mutations. The most common EGFR mutation in glioma is the de2-7EGFR, a 267 amino acid deletion of the extracellular domain (ECD) [7]. It is constitutively active, unable to bind known ligand, cancer specific, and is not detectable in normal tissue [8]. The clinical importance of de2-7EGFR is evident as its expression is closely linked to increased tumor aggression, invasion and poor prognosis for survival [7].

The antibody mAb806 which was raised against the de2-7EGFR, also binds the full length EGFR but only when it is activated through overexpression, mutation or autocrine activity [9,10]. Since the EGFR is not constitutively active in normal tissue, the mAb806 is cancer-specific. Epitope mapping has shown that the mAb806 epitope is located in short cysteine loop of the EGFR (amino acids 311-326) [11].

Recently, several novel glioma-specific missense mutations in the ECD of the EGFR were identified [12]. Two of these mutations, R324L and E330K, are located within or close to the region of the mAb806 binding site. Importantly, a similar corresponding region in ErbB2 has also been found to be mutated [13]. Given the location of these mutations and their proximity to the mAb806 binding site, we sought to understand the function of these missense mutations in NR6 cells as these do not express any ErbB family members. Interestingly, the N-terminus of the de2-7EGFR ECD also starts in this region and contains a free cysteine adjacent to the mAb806 epitope. Using the de2-7EGFR as a model we examined the role of cysteine residues in this region with respect to mutant EGFR activation.

## 2. Results

### 2.1. Expression of the ECD Mutations

The expression of two EGFR ECD mutants (R324L and E330K) was examined in a cell-free system and NR6 cells. The R324L mutation is located within the disulfide loop containing the mAb806 epitope and the E330K mutation is located within an adjacent disulfide loop. Characterization of the A289V has been reported previously [12] and was included as a control in several experiments.

**Figure 1.**
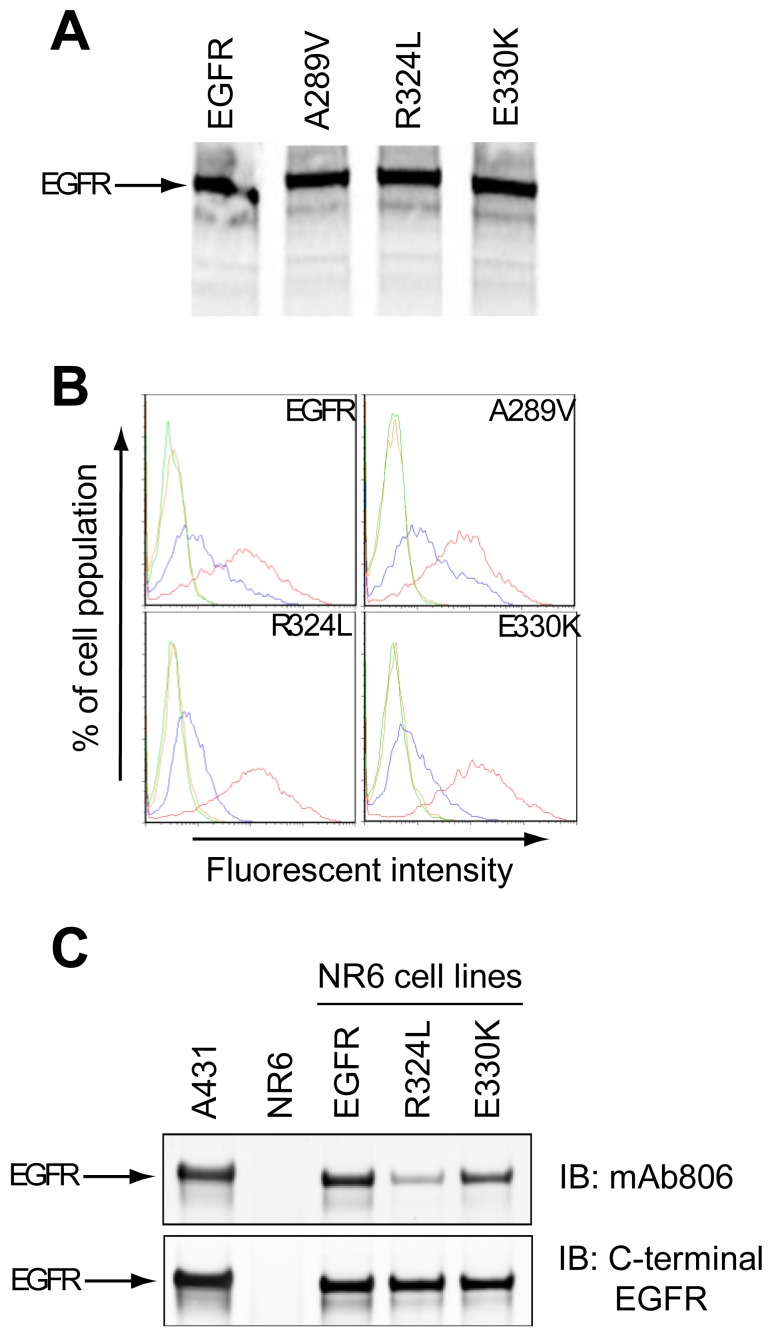
Biochemical characterization of mutant epidermal growth factor receptor (EGFR). (**A**). wtEGFR and mutant EGFR were translated into ^35^S-cysteine labelled proteins in a cell free expression system and labeled proteins detected by autoradiography following SDS-PAGE; (**B**). NR6 cells expressing wtEGFR or mutant EGFR were stained with secondary antibody alone (green), isotype control antibody (orange), mAb528 (red) or mAb806 (blue) and subjected to Fluorescence Activated Cell Sorting *(*FACS) analysis. The representative profiles for each cell line are shown; (**C**). wtEGFR or mutant EGFR cells were grown under serum free conditions, lysed and IP'd using mAb528. Western blot analysis of total EGFR using a mAb806 probe (*upper panel*) or a C-terminal EGFR probe (*lower panel*) are shown. A431 cells that overexpress the EGFR were included as a positive control.

The cell-free translation of mutant EGFR demonstrated that Mw and expression levels of the proteins were similar to the wtEGFR, indicating that the mutations did not affect translation efficiency (Figure 1A). Both the wtEGFR and mutant EGFR were expressed on the cell surface of NR6 cells as determined by Fluorescence Activated Cell Sorting (FACS) (Figure 1B). Since the mAb528 antibody is highly conformation dependant, its ability to recognize the mutants suggests that they are folded correctly. The mAb806 recognized a portion of the overexpressed wtEGFR as expected (Figure 1B), but a greater shift was evident for wtEGFR and A289V than the R324L or E330K mutants, suggesting that the latter two mutations may affect the binding of the mAb806. This was also observed in our Western analyses (Figure 1C) where mAb806 displayed reduced binding especially to the R324L mutation relative to wtEGFR (*upper panel*). Binding of a C-terminal EGFR antibody was unchanged (*lower panel*) when comparing the fully glycosylated upper EGFR band. Thus the R324L and E330K mutations are expressed on the cell surface and have a minor, but reproducible, impact on mAb806 binding.

### 2.2. ECD EGFR Mutations Are Constitutively Active and Retain Response to Ligand

**Figure 2.**
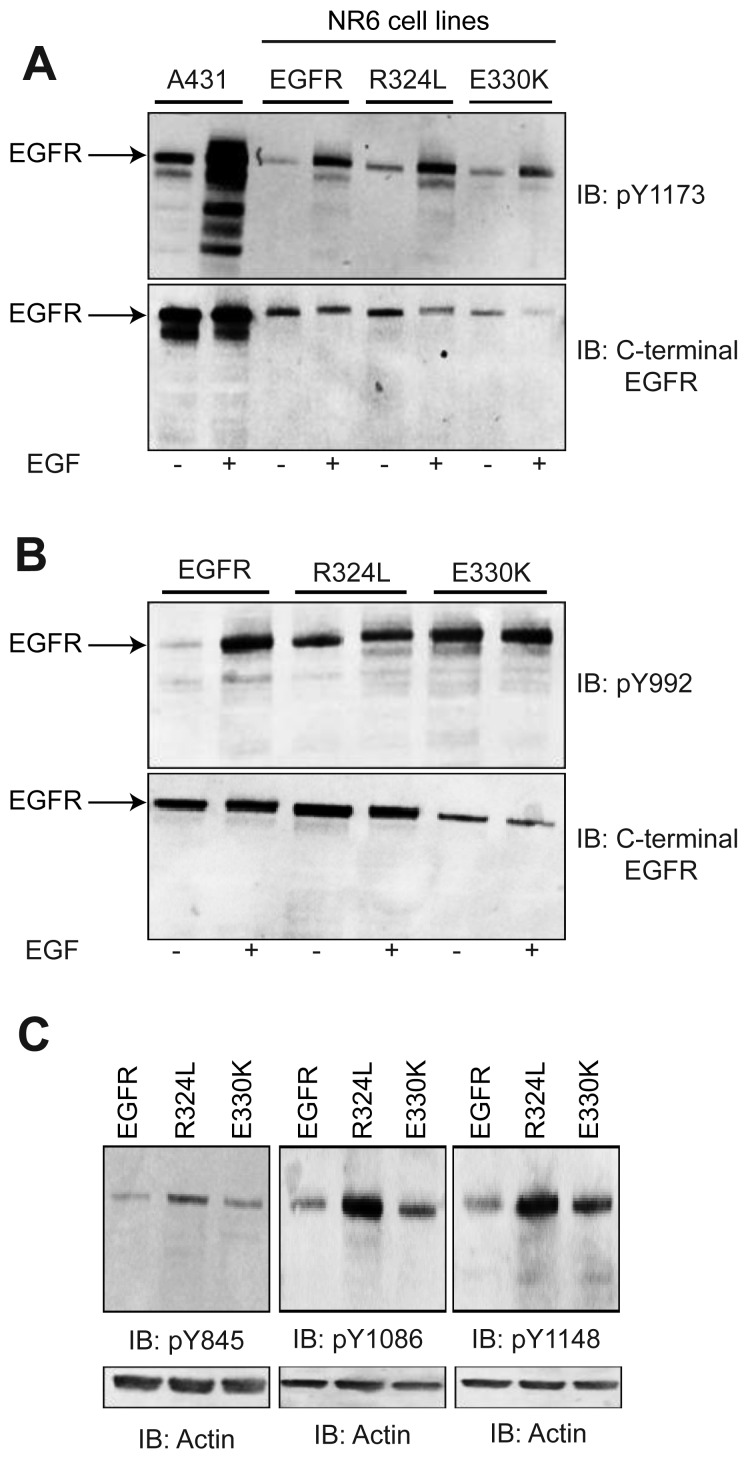
Activation of the EGFR mutants. Whole cell lysates (WCL) from NR6 cells expressing wtEGFR or the R324L and E330K mutants were analyzed by Western blot at multiple phosphorylation sites without or with EGF stimulation. (**A**). pY1173 analysis (*upper panel*) and C-terminal EGFR (*lower panel*). A431 cells that overexpress the EGFR were included as a positive control; (**B**). pY992 blot (*upper panel*) and C-terminal EGFR (*lower panel*); (**C**). Basal phosphorylation status of pY845 (*left*), pY1086 (*middle*) and pY1148 (*right*) blots and their corresponding actin loading controls. In all cases representative results from multiple repeats are shown.

To examine if the EGFR mutants were constitutively activated or could respond to ligand, Whole cell lysates (WCL) from transgenic NR6 cells were blotted with a panel of pY EGFR antibodies (Figure 2). Basal phosphorylation of the EGFR (Figures 2A and 2B) was enhanced in the absence of ligand stimulation for both the R324L and E330K mutants slightly at Y1173 (2 fold and 5 fold increases when corrected for total EGFR, respectively) and dramatically at Y992 (3 fold and 10 fold increases, respectively) relative to wtEGFR. Examination of the Y1086 and Y1148 sites also showed enhanced basal phosphorylation for the mutants, particularly R324L, relative to wtEGFR (Figure 2C). However, Y845 basal phosphorylation, a target of Src phosphorylation and not autophosphorylation, was only increased slightly (Figure 2C). EGF stimulation resulted in increased phosphorylation at Y1173 and Y992, the two sites examined, for both R324L and E330K (Figures 2A and 2B). Thus the R324L and E330K mutants are constitutively active and respond to ligand.

### 2.3. Downstream Signaling by EGFR Mutations

**Figure 3.**
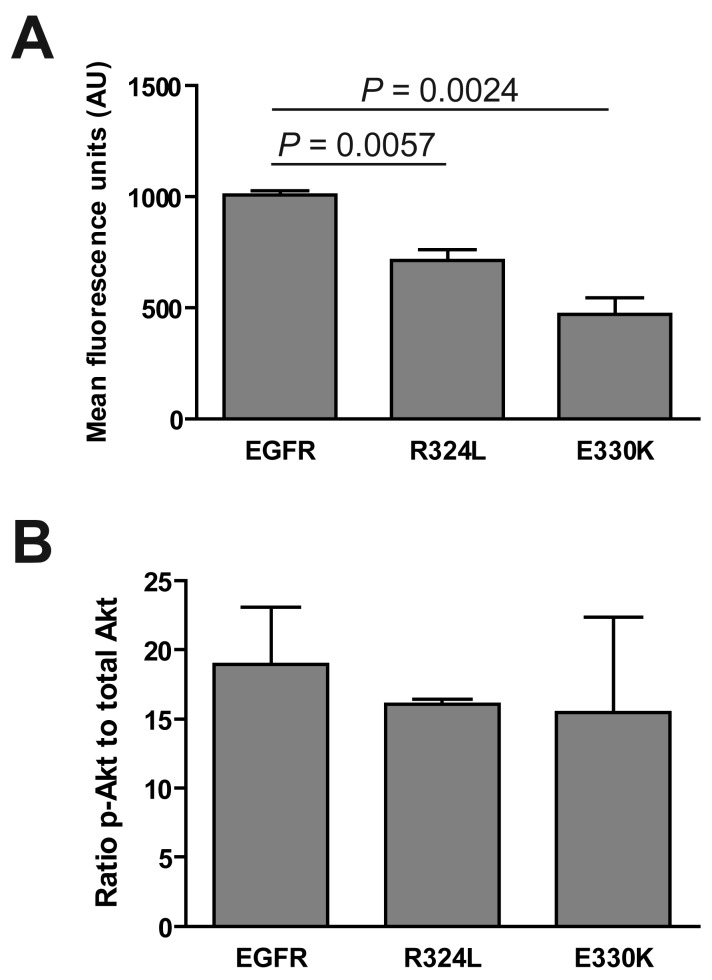
Activation of downstream pathways by mutant EGFR. Transgenic NR6 cells expressing wtEGFR, R324L and E330K were plated under serum free conditions and then lysed and tested by Bioplex for (**A**) pERK1/2 and (**B**) pAkt levels. Data is presented as mean fluorescent intensity ± S.E. or as a ratio of p-Akt to t-Akt ± S.E. of three independent replicates corrected for total Akt protein.

We determined if the EGFR mutations affected the activation of the extracellular signal-regulated kinase (ERK) 1/2 and Akt. Although ERK was phosphorylated in response to EGF stimulation (data not shown), basal levels of pERK were significantly decreased for both the R324L and E330K mutants compared to wtEGFR (*P* < 0.01 for both; Figure 3A). Basal pAkt:total Akt ratios were similar for wtEGFR and mutant EGFR (Figure 3B). These results suggest that these constitutively active EGFR mutants down-regulate pERK1/2 but do not activate Akt.

### 2.4. EGFR Mutations Bestow an *In Vitro* Survival Advantage to NR6 Cells

**Figure 4.**
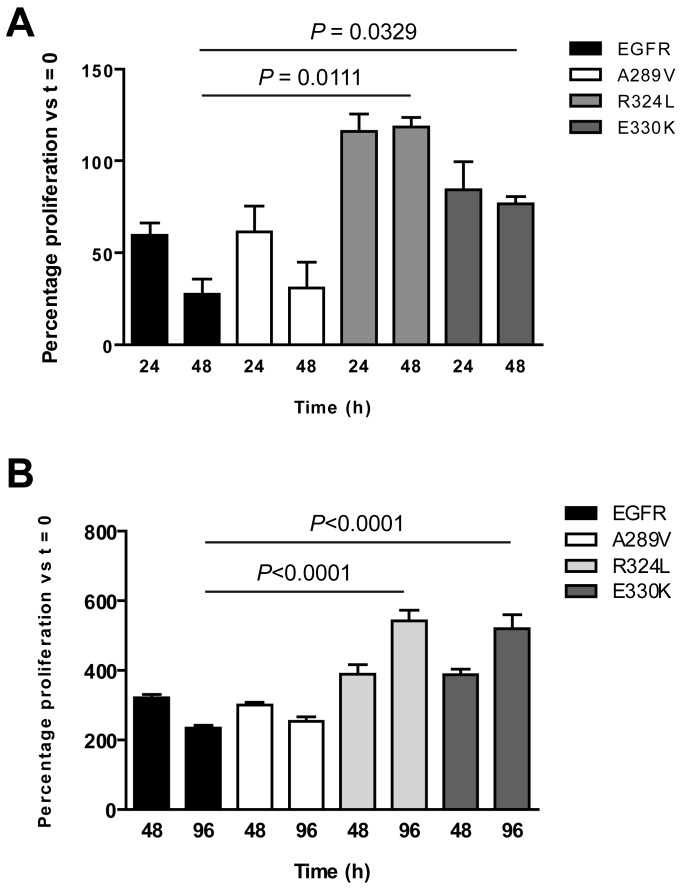
The R324L and E330K mutants show increased cell survival under serum free and 2% FBS conditions. Transgenic cells were cultured under (**A**) serum free conditions for 48 h and (**B**) in media containing 2% FBS for 96 h. Cells were tested for proliferation at various time points using the MTS assay. The data shown is the percentage of cell growth at each time point compared to the 0 h control ± S.E.

The survival of NR6 cells expressing the EGFR mutants was examined under serum-free and low serum conditions at various time points to determine any *in vitro* survival advantages. The NR6 cells expressing R324L showed enhanced proliferation within the first 24 h of culture (Figure 4A). Also, while the number of wtEGFR and A289V NR6 cells decreased substantially over 48 h in serum free conditions, the number of NR6 cells expressing either R324L or E330K remained steady (Figure 4A), indicating a retention of cell viability. When grown in 2% fetal bovine serum (FBS) over 96 h (Figure 4B), the cell numbers for the R324L and E330K mutants continued to increase and were significantly higher than the wtEGFR and A289V cells, which began to die, at the 96 h time-point. These data show that the R324L and E330K also provide a survival advantage to cells in low serum conditions.

**Figure 5.**
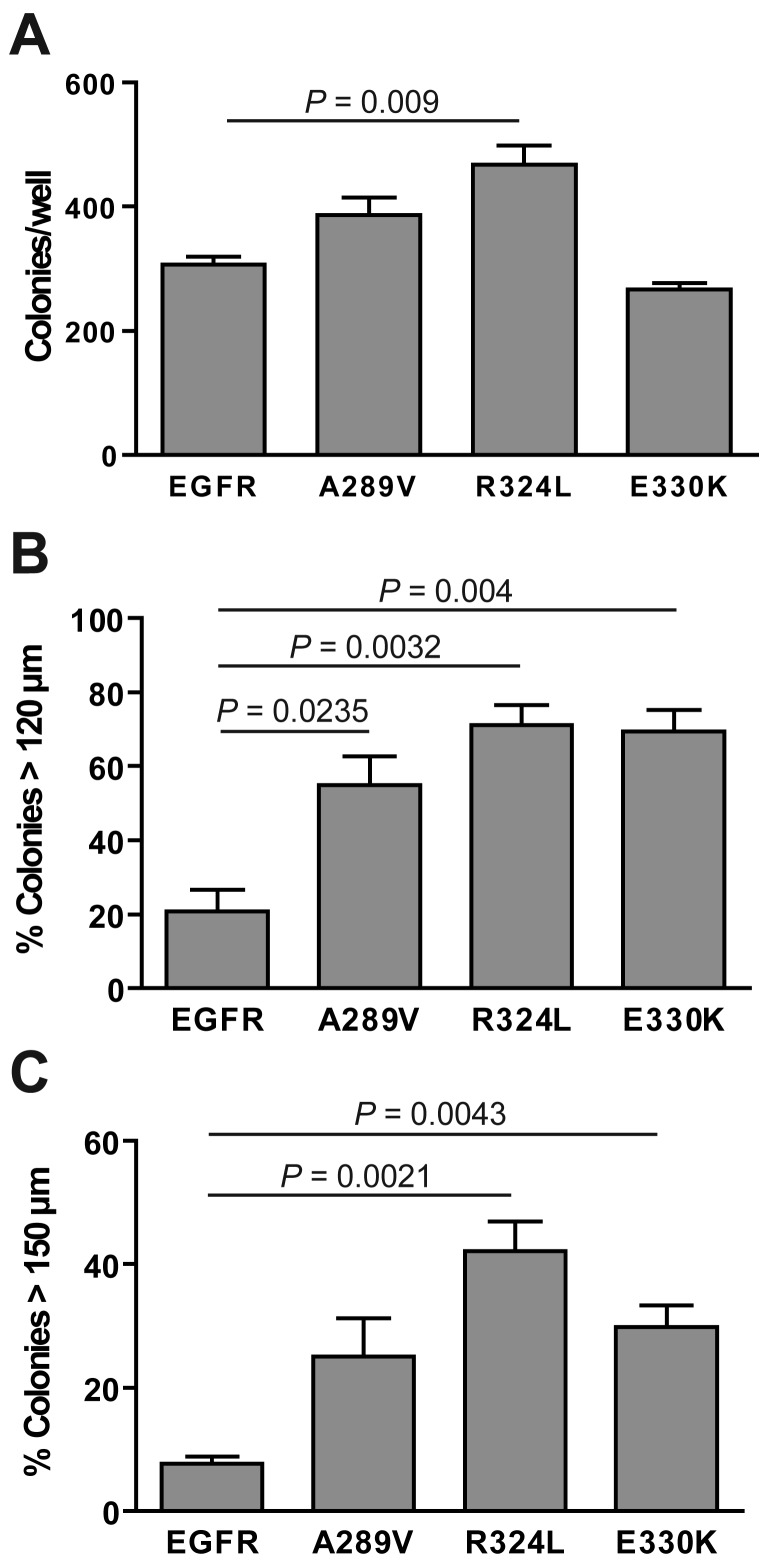
The R324L and E330K mutants demonstrate enhanced transforming activity in anchorage independent growth assays. Transgenic NR6 cells were plated in an agarose matrix for 20 days and stained with MTT. (**A**) Colonies were counted and data graphed as the total colonies per well ± S.E. A random 10–20% sampling of the total numbers was analyzed for the percentage of cells (**B**) over 120 μm or (**C**) over 150 μm in size ± S.E.

The ability of the transgenic NR6 cells to form colonies in anchorage-free conditions was examined (Figure 5). All of the cells tested formed colonies, with the R324L mutant forming significantly more colonies than the other cell lines (*P* = 0.009; Figure 5). The E330K mutant did not form significantly more colonies than wtEGFR-expressing cells. When the data was grouped into the percentage of colonies greater than 120 μm (Figure 5B) and 150 μm (Figure 5C), it was clear that both the mutant EGFR-expressing cells formed colonies that were significantly larger in size than wtEGFR. Thus, the ECD EGFR mutations significantly enhanced *in vitro* survival in serum free conditions and soft agarose when compared with wtEGFR.

### 2.5. EGFR Mutations Increase Tumorgenicity of NR6 cells *In Vivo*

**Figure 6.**
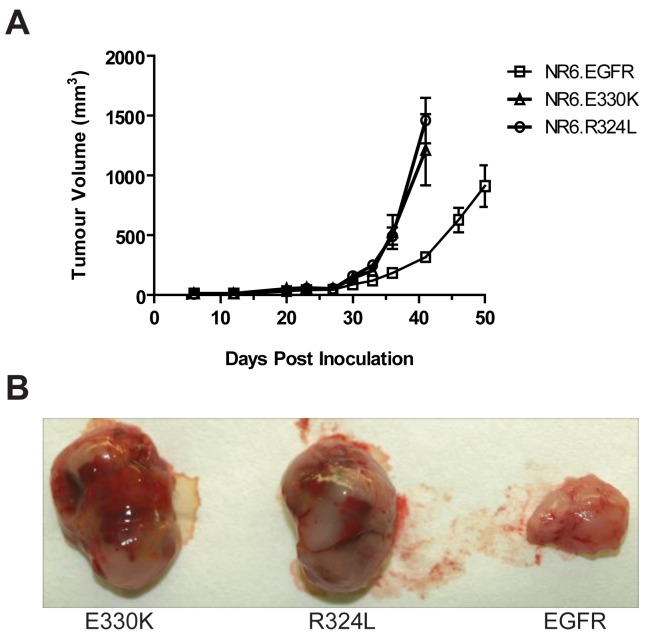
EGFR mutants promote *in vivo* tumor growth. Nude mice were injected subcutaneously into both flanks with transgenic NR6 cells containing wtEGFR, R324L or E330K. (**A**) Growth curves for NR6 cells expressing wtEGFR or R324L or E330K mutants. Data is presented as mean tumor volume ± S.E; (**B**) Gross tumors surgically resected for E330K, R324L and wtEGFR after 40 days.

Transgenic NR6 cells expressing wtEGFR, R324L or E330K were injected s.c. into BALB/c nude mice and tumor development was followed for 50 days. Expression of the EGFR mutations in NR6 cells caused an accelerated growth rate over time compared to the wtEGFR (Figure 6A). The R324L and E330K tumors at day 40 were 4–5 times bigger than that observed for wtEGFR tumors (1.15–1.5 cm^2^ *vs.* 0.25 cm^2^ for the mutant EGFR and wtEGFR respectively). Examination of the tumor appearance after surgical resection revealed that the mutant EGFR tumors were bulbous and heavily vascularized, whereas the wtEGFR tumors were flat, pale and possessed little vascularization (Figure 6B). These results show that the R324L and E330K mutants conferred a significant growth advantage and enhanced tumorgenicity of NR6 cells *in vivo*.

### 2.6. de2-7EGFR Undergoes Reduction-Sensitive Dimerization Utilizing a Free N-Terminal Cysteine

**Figure 7.**
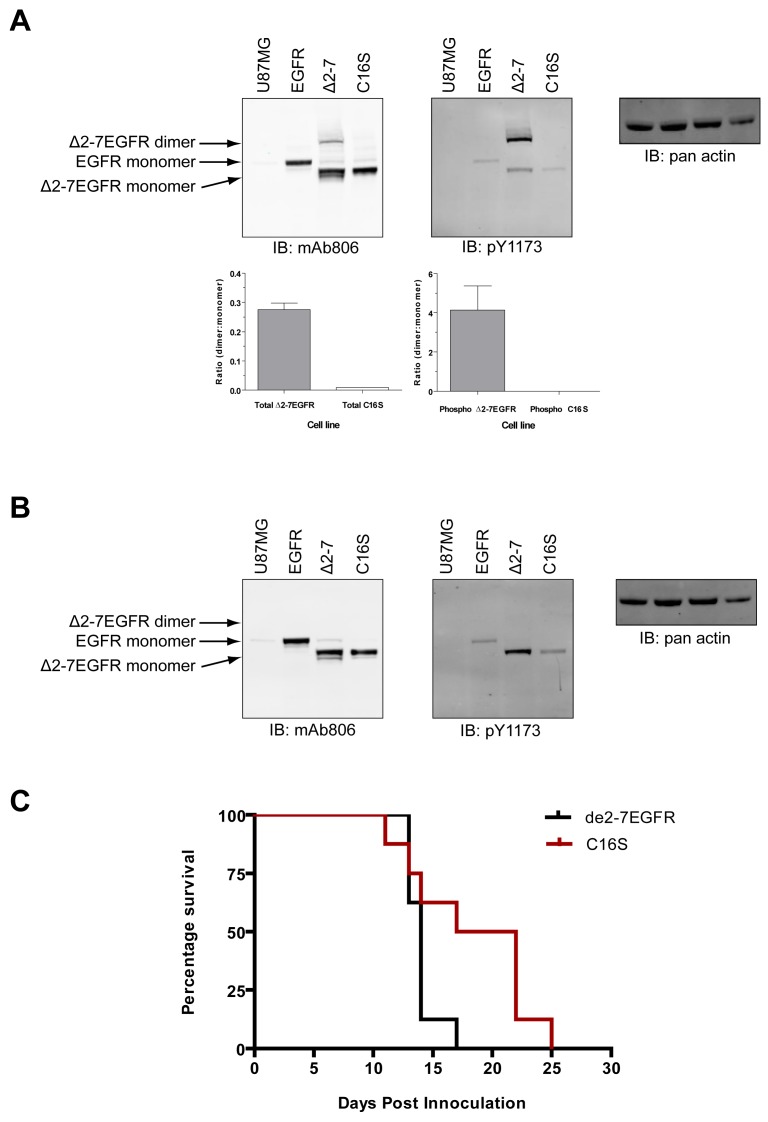
de2-7EGFR forms reduction-sensitive homodimers *in vitro* through its free cysteine residue. Cells were lysed and equal protein probed by Western blot under non-reducing (**A**) and reducing (**B**) conditions for total EGFR/de2-7EGFR by mAb806 blotting (*left hand panels*) and pY1173 (*middle panels*). A pan actin blot was used as the loading control (*right hand panels*). Graphed data of dimer:monomer densitometry ratios ± S.E. for non-reducing mAb806 (*lower left graph*) and pY1173 (*lower right graph*) are depicted under each blot in (**A**). U87MG parental cells (U87MG) and U87MG overexpressing the wtEGFR (EGFR) were included as control for the wtEGFR on the blots. In all cases representative results from multiple repeats are shown (**C**) C16S is less tumorigenic than unmodified de2-7EGFR. Nude mice were injected subcutaneously into both flanks with transgenic U87MG cells expressing either unmodified de2-7EGFR or the C16S variant. Kaplan-Meier survival curves were then determined based on a tumor volume of 1000 mm^3^.

The de2-7EGFR truncation breaks the EGFR cysteine pairing of C295-C307 and removes the C295 residue, creating an unpaired cysteine at C307 (identified by us here as C16 in the mature de2-7EGFR sequence). This cysteine maps to the same region as our missense mutations. In addition, it is possible that this free cysteine could lead to dimerization and activation of de2-7EGFR via formation of an intermolecular disulfide bond. To investigate this, we mutated the C16 to serine (C16S) in the de2-7EGFR. Both the de2-7EGFR and the C16S were successfully expressed in U87MG cells (Figure 7). Under non-reducing conditions (*upper left panel*, Figure 7A), a de2-7EGFR dimer was clearly observed but was virtually absent in the C16S mutant even when the blots were overexposed (*left panel*, Suppl. Figure 1) proving that de2-7EGFR dimerization requires this free cysteine. Quantification of the dimer: Monomer densitometry ratios for total protein (*lower left graph*, Figure 7A) showed the de2-7EGFR dimer formed a high proportion of the total species (∼25%). As expected, the dimer disappeared in the reducing mAb806 blot (*left panel*, Figure 7B). When non-reducing blots were probed with the pY1173 EGFR antibody, the de2-7EGFR dimer was strongly phosphorylated whilst no phosphorylated dimer was detectable for C16S (*upper middle panel*, Figure 7A). Densitometry analyses confirmed that the de2-7EGFR dimer was the active form of the receptor as it was 4.1 times more intense than the monomer (*lower right graph*, Figure 7A). Reduction of the samples and probing for pY1173 showed that the overall phosphorylation of the C16S mutant was markedly decreased compared to the unmodified de2-7EGFR (*middle panel*, Figure 7B). These data demonstrate that the free cysteine created as a consequence of the de2-7EGFR mutation is critical for the formation and stabilization of the active de2-7EGFR dimer.

### 2.6. The C16S Modification Displays a Reduced Tumorigenicity *In Vivo*

In order to see if the ablation of the free cysteine in de2-7EGFR has any *in vivo* consequences, U87MG cells expressing unmodified de2-7EGFR or the C16S variant were injected s.c. into BALB/c nude mice and tumor development followed. The survival of each population was then compared by Kaplan-Meier survival analysis (Figure 7C). Animals injected with C16S variant survived significantly longer (*P* < 0.05) than those injected with unmodified de2-7EGFR, demonstrating that the reduction observed in *in vitro* activity translates into a reduced tumorigenicity *in vivo*.

## 3. Discussion

Glioma specific single point missense mutations in the cysteine rich region of EGFR near the mAb806 epitope lead to autoactivation and enhanced tumorigenicity. There were pronounced differences with respect to the tyrosine phosphorylation pattern seen for each mutant and neither R324L nor E330K strongly activated pY1173, previously identified as the key phosphorylation site for activity of the de2-7EGFR [14]. This suggests that clonal selection within the tumor might result in EGFR mutations with differing signaling properties. The preponderance of ECD mutations in glioma, in contrast to the kinase mutations that dominate lung cancer, further supports the idea that mutations arise in a context specific manner. Both mutants, but especially E330K, showed increased phosphorylation at Y992. Phosphorylation at this site reduces ligand-induced receptor endocytosis, increasing the lifetime of the activated receptor in the plasma membrane and therefore enhancing signaling capacity [15]. Recently, an Akt-independent PLCγ-PKCα-mTOR pathway was identified in glioma [16]. It is possible that our mutations preferentially activate this pathway and may partially explain the lack of Akt activation. The decrease in pERK mediated by the R324L and E330K mutant is consistent with recent findings showing pERK is negatively correlated with de2-7EGFR [17].

Various reports suggest that the de2-7EGFR only forms transient homodimers [14,18,19]. An early publication showing robust dimerization of de2-7EGFR has not been reproduced [20] and a second paper reporting significant dimerization used non-physiological conditions including a lengthy purification [21]. However, de2-7EGFR homodimers must exist as auto-phosphorylation of the EGFR always occurs in *trans* through the formation of an asymmetric dimer [22]. Indeed, we used differentially tagged de2-7EGFR molecules to demonstrate this transient interaction [18]. Using the drug iodoacetamide during lysis to protect disulfide bonds, we could reproducibly show significant levels of dimerized de2-7EGFR. Importantly, detection of the de2-7EGFR dimer in our system was totally dependent on the presence of the free cysteine at position 16. The previous confusion about the presence or absence of de2-7EGFR dimers seems to be caused by the use of differing techniques. Detection of these dimers requires the addition of reagents that protect the intermolecular disulfide bond as it appears extremely labile during cell lysis. Finally, the intense phosphorylation of the de2-7EGFR dimer, especially when the phosphorylated receptor is compared to total receptor (Figure 7), clearly demonstrates that this is the active conformation.

**Figure 8.**
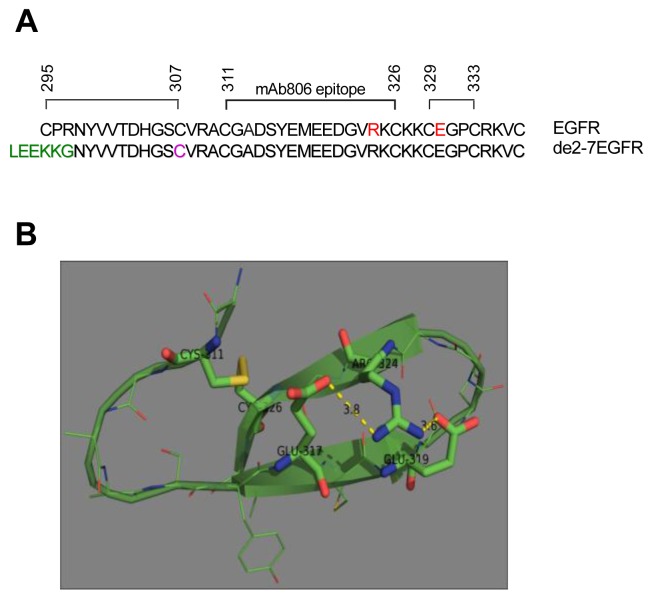
(**A**). Alignment of amino acids 295-337 of the immature EGFR protein with amino acids 1-46 of the mature de2-7EGFR protein. The disulfide bonds (black lines), mAb806 epitope and position of two EGFR ECD mutations studied in this paper (red) are shown. A 267 amino acid truncation removes the C295 from the EGFR structure and results in a free C16 residue in the mature de2-7EGFR protein (pink) as well as a unique 6 amino acid N-terminal sequence (green); (**B**). Three dimensional structural model of module 7 incorporating the R324L mutation. R324 lies within the module 7 loop spanning amino acids 311-326 in Domain II of EGFR. The R324L mutation will abolish salt bridges (dashed lines) with E317 and E319 which stabilize the loop, leading to greater loop flexibility and significant strain on the C311-C326 bond (yellow). Analysis utilized the structure of ligand bound EGFR extracellular domain described previously [3]. The figure was prepared with PyMol v.1.2r2 software (Schrodinger, LLC).

Unfortunately, while we were able to show that the missense mutations were present as dimers using the techniques described here (data not shown), we were unable to definitively determine their nature because of their low abundance and the presence of alternative inactive dimers as previously reported [23]. Also, the ability to mutate the exact cysteine residue in the de2-7EGFR made the definitive experiments relatively straight-forward in this receptor. Obviously this approach is not possible in full-length receptors. We have postulated that the region around mAb806 must be locally misfolded during receptor activation [10]. As noted, the R324L mutation is found within the mAb806 epitope and the E330K mutation is in the adjacent cysteine loop (Figure 8A). Our results show that these mutations cause conformational changes that enhance receptor activation in the absence of ligand. The R324L mutation abolishes two salt bridges with adjacent E317 and E319 residues. These salt bridges appear to stabilize the C311-C326 disulfide loop (Figure 8B). Their removal should increase the flexibility and dynamic behavior of the loop and place strain on the C311-C326 disulfide bond. This would make the bond more susceptible to breaking and possibly prevent its formation, leaving a free cysteine for intermolecular bonding as for the de2-7EGFR. Indeed, the cysteine at 311 is only a few amino acids away from the free cysteine in de2-7 EGFR which is cysteine 307 (or C16 in the de2-7EGFR). Molecular modeling has suggested that a G336R ECD mutation found in zebra fish EGFR also disrupts a nearby disulphide bond resulting in the formation of disulphide linked homodimers [24]. Taken together these results suggest a general mechanism of activation, whereby mutations in this cysteine rich region lead to disruption of disulfide bonds which become available for stabilizing dimer interactions.

Mutations in other RTKs have been shown to create free cysteines that mediate receptor dimerization. The ECD mutations W290G or T341P within FGFR-2, which are directly linked to the initiation of Crouzon and Pfeiffer syndromes, release a free cysteine by disrupting a nearby intramolecular disulfide bond, triggering intermolecular bonding, activation and conferring tumorigenic properties in NIH3T3 cells [25]. Other examples of free cysteine-linked activation include the R129C ECD erythropoietin receptor mutation [26,27], C342Y ECD FGFR-2 mutation [28] and the c-RET MEN-2A ECD germline mutation C634R [29]. All of these mutants are dimerized, constitutively active and directly linked to induction of various diseases or cancers.

## 4. Experimental

### 4.1. EGFR Numbering

Through-out the paper we have numbered EGFR based on the initiating methionine, as per the original mutation paper. However, numbering of phosphospecific antibodies is based on the mature EGFR as per common use. The difference between the two numbering formats is 24 amino acids.

### 4.2. Antibodies and Reagents

mAb528 and mAb806 were produced and purified at the biological production facility at the Ludwig Institute for Cancer Research, Melbourne, Australia. The anti-C-terminal EGFR mAb R13 was from BD Biosciences (San Jose, CA). The anti-C-terminal EGFR polyclonal antibody 1005 and the Protein A/G beads were from Santa Cruz Biotechnology (Santa Cruz, CA). All anti-phosphorylated EGFR antibodies (pY845, pY992, pY1068, pY1086, pY1148 and pY1173), as well as the anti-mouse IgG conjugated to Alexa Fluor 680, were from Invitrogen (Carlsbad, CA). The anti-phosphorylated Y1173 EGFR (clone 53A5) and the anti-β-actin polyclonal were from Cell Signaling (Danvers, MA). The anti-pan actin mAb ACT05 was from ThermoFisher Scientific (Waltham, MA). Horse radish peroxidase (HRP)-conjugated anti-mouse or anti-rabbit secondary antibodies were from Millipore (Billerica, MA). Anti-mouse IgG whole molecule-R-phycoerythrin (PE) conjugated secondary antibody, human recombinant EGF and iodoacetamide were from Sigma Aldrich (St Louis, MO). Anti-rabbit IgG polyclonal antibody conjugated to IRDye800 was from LiCor Biosciences (Lincoln, NE). Site directed QuikChange mutagenesis XL kit was from Stratagene (La Jolla, CA). All primers were synthesized at Sigma Aldrich or at Micromon (Monash University, Clayton).

### 4.3. Cell Lines

The human glioma cell line U87MG (HTB-14) and the human epidermal carcinoma cell line A431 (CRL-1555) were sourced from the American Type Culture Collection (ATCC, USA). The NR6, U87MG.Δ2-7 and U87MG.EGFR cell lines have been described by us previously [30]. The NR6.EGFR, NR6.A289V, NR6.R324L and NR6.E330K cell lines were constructed as follows; mutations were introduced into the EGFR ORF, subcloned in pGEM-4Z (Promega), by site-directed mutagenesis using the primer sets detailed in Suppl. Table 1. Next, the mutant EGFR ORF's were cloned into the pBABEpuro retroviral vector and transfected into a viral packaging cell line using Fugene (Roche), with subsequent viral supernatants used to infect NR6 cells. Transfected cells were selected in the presence of puromycin (2 μg/mL) and surviving cells FACS sorted for high EGFR expression using mAb528.

The U87MG.Δ2-7_C16S_ cell line was constructed using primers detailed in Suppl. Table 1 and site-directed mutagenesis to incorporate a Cys-Ser mutation at amino acid position 16 (the equivalent amino acid is C307 in wtEGFR) within the de2-7EGFR ORF also using the pBABEpuro retroviral vector. Cells were FACS sorted with mAb806 to obtain a surface expression level similar to U87MG.Δ2-7. All cell lines were maintained in DMEM/F12 containing 5% FBS, 2 mM Glutamax and 100 units of penicillin/streptomycin in the presence of suitable selective drug.

### 4.4. *In Vitro* Cell-Free Protein Expression

EGFR-related cDNA's were translated into ^35^S-cysteine labelled proteins from pGEM-4Z using the TNT-coupled reticulocyte lysate kit according to manufacturer's protocol (Promega, Madison, WI).

### 4.5. Fluorescence Activated Cell Sorting (FACS) Analysis

2–3 × 10^5^ cells were incubated with the indicated antibody at 10 μg/mL in 0.1% human serum albumin/PBS (HSA-PBS) for 1 h at 4 °C and then incubated with 1:20 anti-mouse IgG-PE secondary antibody in HSA-PBS at 4 °C for 30 min. Cells were resuspended in FACS fix solution (PBS; 3.2% D-glucose; 1% formaldehyde) and run on a Guava flow cytometer (Millipore).

### 4.6. Immunoprecipitation and Western Blotting

Immunoprecipitation of EGFR from 1–3 × 10^6^ transgenic NR6 cells was carried out essentially as previously described [30] using 1 μg/ml of mAb528 to capture the EGFR. Bead pellets after washing were resuspended in reducing LDL loading buffer containing 100 mM DTT. Reducing SDS-PAGE and western analysis were essentially as previously described [30].

### 4.7. MTS Cell Growth Assays

Transgenic NR6 cells expressing wtEGFR or mutant EGFR were seeded in triplicate at 5000 cells/well in 96 well plates and allowed to adhere overnight. Cells were washed in serum-free medium after which cells were incubated in serum-free medium at 37 °C, 5% CO_2_ for 48 h. At 0 h, 24 h and 48 h, MTS was added to the wells. After 3 h of incubation at 37 °C, the assay was read at 490 nm using a FLUOstar Optima plate reader (BMG Labtech, Offenburg, Germany).

### 4.8. Anchorage-Independent Growth Assay

Using 6-well plates, a bottom layer of 0.8% low melting agarose (LMA) was made by combining equal quantities of melted 1.6% LMA and 2 × media (DMEM-F12; 10% FBS; 4 μg/mL puromycin). The top layer of 0.4% LMA was made by combining equal volumes of melted 0.8% LMA and 2 × media containing the transgenic cells, at 1000 cells/well. Plates were incubated at 37 °C, 5% CO_2_ overnight, then 500 μL/well of media was added and plates incubated for 20 days. Wells were washed twice with PBS and 500 μL of 1 mg/mL MTT was added for 24 h after colonies photographed and counted.

### 4.9. Dimerization Analysis of de2-7EGFR

This was conducted using an established protocol [31] with minor modifications. Confluent cells were harvested, washed twice in ice cold PBS (pH 8.0) containing 10 mM iodoacetamide and lysed in ice cold RIPA buffer [50 mM Tris (pH 7.5); 150 mM NaCl; 5 mM EDTA; 0.5% sodium deoxycholate; 0.05% SDS; 10 mM NaF; protease inhibitor cocktail set 1 (Calbiochem, USA); 200 μM Na_3_VO_4_; 10 mM iodoacetamide] for 10 min on ice. Lysates were sonicated and clarified at 21,100 × g for 20 min at 4 °C. Equal protein amounts, as determined by BCA assay (Sigma), were resuspended in either non-reducing Laemmli buffer or reducing Laemmli buffer containing 0.8 M of β-mercaptoethanol and analyzed by SDS-PAGE and western blotting.

### 4.10. Bioplex Assay

Transgenic NR6 cells plated at 2 × 10^5^ cells/well were serum-starved for 24 h prior to stimulation with EGF at a final concentration of 100 ng/mL for 15 min, at 37 °C. Whole cell lysates (WCL) were prepared using Bioplex cell lysis buffer (BioRad, Hercules, CA) and pAkt and pERK1/2 were quantified according to the manufacturer's instructions.

### 4.11. *In Vivo* Xenograft Models

2 × 10^6^ transgenic NR6 cells in PBS were injected subcutaneously into both flanks of six week old female BALB/c nude mice. Tumors were monitored and measured as previously outlined [30]. This research project was approved by the Animal Ethics Committee of the Austin Health, Heidelberg, Australia.

## 5. Conclusions

Our previous studies with mAb806 have clearly identified a critical role for the cysteine-rich region around amino acids 311-326 in EGFR activation [10,11]. We now show that glioma-associated missense mutations in this region lead to ligand independent activation of EGFR. Significantly, a recent report identified activating mutations in the same cysteine rich region of ErbB4 in melanoma [32]. These studies and our current observations strongly suggest that local misfolding in this cysteine rich region is important to EGFR activation. Ongoing studies into the nature of this local misfolding, and the potential role of transient disulfide bond cleavage, should be informative with respect to the mechanisms associated with activation of the ErbB family.

## Supplementary Files

Supplementary File 1 PDF-Document (PDF, 367 KB)

Supplementary File 2 PDF-Document (PDF, 203 KB)
