# Supplementary material for: Glioma Specific Extracellular Missense Mutations in the First Cysteine Rich Region of Epidermal Growth Factor Receptor (EGFR) Initiate Ligand Independent Activation
Source: Cancers (Basel). 2011 Apr 18;3(2):2032–49. doi: 10.3390/cancers3022032 (PMC3757403; doi:10.3390/cancers3022032)
Supplement: Supplementary File 1 — PDF-Document (PDF, 367 KB) [file cancers-03-02032-s001.pdf]

**Supplementary Table 1:** Primer sequences used for introduction of EGFR ECD mutations

| Primer | Sequence (5' → 3')* |
|---|---|
| A289V For | GGC AAA TAC AGC TTT GGT GTC ACC TGC GTG AAG AAG TG |
| A289V Rev | CAC TTC TTC ACG CAG GTG ACA CCA AAG CTG TAT TTG CC |
| R324L For | GAG GAA GAC GGC GTC CTC AAG TGT AAG AAG TGC |
| R324L Rev | GCA CTT CTT ACA CTT GAG GAC GCC GTC TTC CTC |
| E330K For | GCA AGT GTA AGA AGT GCA AAG GGC CTT GCC GCA AAG |
| E330K Rev | CTT TGC GGC AAG GCC CTT TGC ACT TCT TAC ACT TGC |
| C16S For | GAT CAC GGC TCG AGC GTC CGA GCC T |
| C16S Rev | AGG CTC GGA CGC TCG AGC CGT GAT C |

* Underlined sequence depicts the mutated nucleotide
