# Supplementary material for: Glioma Specific Extracellular Missense Mutations in the First Cysteine Rich Region of Epidermal Growth Factor Receptor (EGFR) Initiate Ligand Independent Activation
Source: Cancers (Basel). 2011 Apr 18;3(2):2032–49. doi: 10.3390/cancers3022032 (PMC3757403; doi:10.3390/cancers3022032)
Supplement: Supplementary File 2 — PDF-Document (PDF, 203 KB) [file cancers-03-02032-s002.pdf]

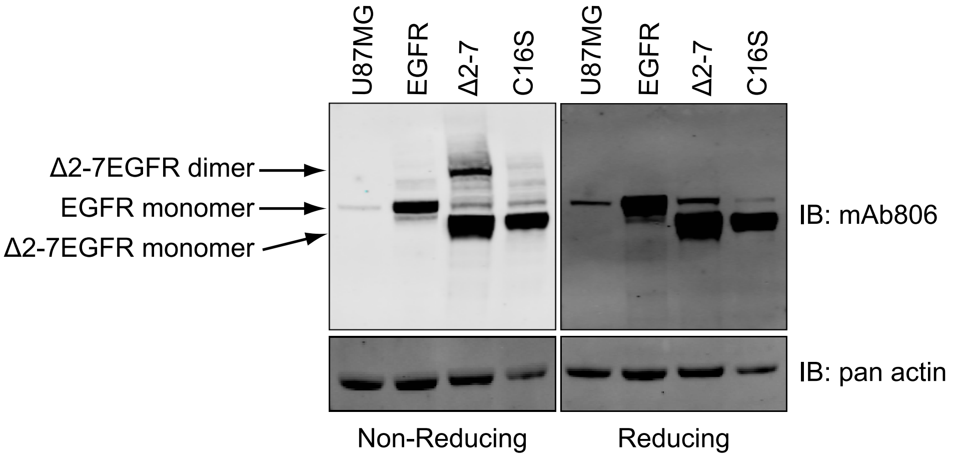

Supplementary Figure 1: Overexposed mAb806 blots from Fig 7 (depicting total EGFR and total de2-7EGFR)
